# Prevalence of Mouse Mammary Tumor Virus-Like Virus in Breast Milk and Associated Factors of Exposure Among Healthy Nursing Women in Morocco

**DOI:** 10.7759/cureus.87047

**Published:** 2025-06-30

**Authors:** Abha Cherkani Hassani, Meriem Slaoui, Bouchra Benfathallah, Imane Ghanname, Rachid Razine, Mohammed Attaleb, Mohammed El Mzibri, Nezha Mouane

**Affiliations:** 1 Laboratory of Analytical Chemistry and Bromatology, Faculty of Medicine and Pharmacy of Rabat, Mohammed V University in Rabat, Rabat, MAR; 2 Laboratory of Histology, Embryology, and Cytogenetics, Faculty of Medicine and Pharmacy of Rabat, Mohammed V University in Rabat, Rabat, MAR; 3 Laboratory of Biochemistry and Molecular Biology, Faculty of Medicine and Pharmacy of Rabat, Mohammed V University in Rabat, Rabat, MAR; 4 Pharmacology, Mohammed V University in Rabat, Rabat, MAR; 5 Laboratory of Biostatistics, Epidemiology, and Clinical Research, Faculty of Medicine and Pharmacy of Rabat, Mohammed V University in Rabat, Rabat, MAR; 6 Biology and Medical Research Unit, National Center for Energy, Science, and Nuclear Techniques, Rabat, MAR; 7 Department of Pediatrics, Hepatology, Gastroenterology, and Nutrition, University Children’s Hospital Ibn Sina, Rabat, MAR; 8 Department of Pediatrics, International University of Rabat, Rabat, MAR; 9 Unit of Training and Research in Nutrition and Food Science, Faculty of Medicine and Pharmacy of Rabat, Mohammed V University in Rabat, Rabat, MAR

**Keywords:** breast milk, healthy women, mmtv-like virus, morocco, oncovirus

## Abstract

Background: The mouse mammary tumor virus (MMTV) has been identified as potentially oncogenic, and its presence in milk was reported and discussed. However, with respect to breast cancer etiology, the transmission routes for these viruses are not known. In this context, the objective of this study is to assess the presence of MMTV-like virus in breast milk and to explore the associated factors of its presence among healthy lactating women in Morocco.

Methods: In this prospective study, milk samples were collected from 44 lactating women recruited from the Souissi Maternity Hospital of Rabat in Morocco. MMTV-like DNA was identified by polymerase chain reaction amplification using specific primers targeting the *env* gene.

Results: The prevalence of the MMTV-like virus was 45.5% (20/44). Statistical analysis revealed a significant association with the use of henna. It seems that it increases the risk of the presence of MMTV-like virus in breast milk. The hypothesis could be the possible contamination of henna sold in bulk in local markets by urine and feces of mice during storage.

Conclusion: Nevertheless, these results need to be confirmed by multicenter studies to thoroughly investigate the impact of other factors and the potential contamination of henna with the virus through urine and feces of mice during storage.

## Introduction

For many years, the hypothesis that a homologous retrovirus to mouse mammary tumor virus (MMTV) is involved in the etiology of breast cancer in humans has fascinated the scientific community, and many studies have been carried out in several developed and developing countries to assess the presence of MMTV-like sequences in breast tissue in women with breast cancer [1,2]. MMTV, firstly reported by Bittner in 1936 as the agent involved in mouse mammary carcinogenesis [3], was suspected as a potential agent for human disease. Thereafter, various studies have been conducted to investigate the association between MMTV-like virus and breast cancer development, and reported results were controversial [4,5]. Some studies have reported the presence of MMTV-like virus in human breast cancer samples [6,7], whereas others reported the absence of MMTV-like DNA in both breast cancer and normal mammary tissues [8-10], maintaining the enigma of the potential role of MMTV-like virus in breast cancer development. Notably, scientific evidence has clearly shown that the MMTV is transmitted in mice either vertically through germ cells or horizontally through breast milk [5,11,12].

In 1971, it was reported that breast milk from healthy women at high risk for breast cancer contained viral particles, visible by electron microscopy, that are morphologically similar to MMTV [13], with reverse transcriptase activity such as oncogenic retroviruses [14].

The presence of MMTV-like virus in milk has been largely reported and discussed. Moore et al. have noted that MMTV-like virus prevails in milk from North American women with a familial history of breast cancer (60%) as compared to North American women with no familial breast cancer history (5%) [13]. Of particular interest, Etkind et al. have reported the presence of viral particles of MMTV-like virus in the milk of women with breast cancer and in the breast tissue of women with a family history of breast cancer [15].

In a study by Nartey et al. [16], two populations were assessed. Among the reference group, which included 92 healthy nursing women, the presence of MMTV-like gene sequences in breast milk was 7.61%. In the biopsy group, 73 women with breast cancer risk and having undergone or were scheduled to have a breast biopsy were included, of which 20.55% had MMTV-like gene sequences in their breast milk. The authors highlighted those results as a foreign origin of the infection and suggested one possible avenue for viral transmission in humans.

In Morocco, a retrospective study conducted in 2014 among 42 Moroccan patients with breast cancer confirmed the presence of MMTV-like sequences in 57.14% cases of breast carcinomas and 33.3% of matched normal breast tissues, suggesting the probable causal role of MMTV-like sequences in breast cancer development [17].

The present study is the first conducted in Morocco and aims to investigate the presence of MMTV-like DNA in breast milk from healthy nursing women as well as to explore its association with several factors, including demographic parameters and maternal anthropometry, and dietary and cosmetic habits.

## Materials and methods

Study design and population

It is a cross-sectional study conducted at the Souissi Maternity Hospital of Rabat in Morocco among healthy nursing women. The inclusion criteria were healthy women who gave birth to healthy newborns with normal birth weight at sampling time-point and accepted to participate in the CONTAMILK pilot study [18].

The exclusion criteria were women with chronic diseases (diabetes, gestational hypertension, asthma, etc.), fever, infections, metabolic diseases, diseases of the breast or central nervous system, malnutrition, and maternal allergy.

Data and sample collection

A face-to-face interview was conducted with each participant by the same interviewer at the time of sample collection, to gather relevant data regarding socio-demographics (residence place, age, level of education, etc.), anthropometric data (body weight before and during pregnancy and height of the mother), clinical data (health status, parity, lactation history, etc.), and other personal habits.

A food frequency questionnaire was used to assess the dietary habits and to collect data from the nursing mothers about the consumption of several foodstuffs to identify the potential sources of MMTV contamination. Further details about the questionnaire, the dietary habits, and the frequency of consumption of foodstuffs were reported in our previous study [18].

Milk samples from lactating women meeting the study’s eligibility criteria were collected. Following proper hygiene procedures, approximately 10 mL of colostrum was manually expressed using a sterilized breast pump between the second and fifth day of postpartum. Samples were transferred into nitric acid-prewashed polyethylene tubes, transported in ice-cooled containers, and stored at -70 °C until analysis. No formal sample size calculation was performed. The number of samples collected was determined based on participants’ availability, voluntary consent, and sufficient colostrum volume, in alignment with the pilot nature of the study.

DNA extraction

Genomic DNA extraction from breast milk samples was performed by the conventional method of alkaline lysis using phenol/chloroform [19]. Briefly, 2 ml of milk was centrifuged at 13,000 g for 10 minutes, and the supernatant was removed. Then, 600 μl of lysis buffer containing proteinase K at 10 mg/ml was added. After an overnight incubation at 42°C, 600 µl of phenol-chloroform was added. After homogenization of the solution by vortex and incubation for 20 minutes at 50°C, the solution was centrifuged for 15 minutes at 13,000 g. Subsequently, the upper phase was collected in a sterile tube. DNA was precipitated with 2/5 volumes of 7.4M ammonium acetate and 2 volumes of 100% ethanol, followed by incubation at -20°C and centrifugation at top speed (13,000 relative centrifugal force). DNA was then re-suspended in 20 µl of DNAse/RNAse-free distilled water. The extracted DNA was stored at 4°C for three hours, and then at -20°C until use. The efficiency of DNA extraction was assessed by human β-globin gene amplification using PC04 and GH20 primers (Table 1).

**Table 1:** List of primers used for polymerase chain reaction amplification and DNA sequencing. F: forward primer; R: reverse primer; pb: base pair.

	Primer	Fragment generated size	Sequence	T° C
β-globin	GH 20 (F)	268 pb	5’ GAAGAGCCAAGGACAGGTAC 3’	65
	PC 04 (R)		5’ CAACTTCATCCACGTTCACC 3’	
MMTV-like detection	489 F	171 pb	5′-ACCAGGGGGTGAGTTTTTCT-3′	57
	659 R		5′-CCCATCCTGCYTCATACCAT-3′	

Detection of MMTV-like viral sequences in breast milk samples

MMTV-like detection was performed by polymerase chain reaction (PCR) using MMTV659R/489F primers to amplify a fragment of 171 bp highly conserved in the *env* gene encoding the viral coat protein (Table 1) [17]. Amplification reaction was performed in a total volume of 25 µl containing 0.4 µM of each primer, 200 µM of each deoxynucleoside triphosphate (dNTP), 0.5 units Taq DNA polymerase (Promega, Charbonnières-les-Bains, France), and 3 µl of DNA sample in 1x Taq polymerase buffer.

All PCR amplifications were done in a GeneAmpR PCR System 9700 thermal cycler (Applied Biosystems, Foster City, CA). Reaction mixtures were first denatured at 94°C for five minutes. Then, 40 cycles of PCR were performed with denaturation at 94°C for 60 seconds, primer annealing at 60°C for 60 seconds, and DNA extension for 90 seconds at 72°C. At the end of the last cycle, the mixtures were incubated at 72°C for 10 minutes. For every reaction, MMTV DNA and sterile water were used as positive and negative controls, respectively. PCR products were analyzed by electrophoresis on 2% agarose gels, followed by staining with ethidium bromide (10 mg/ml).

Statistical analysis

Statistical analyses were performed using both Microsoft Office Excel (Microsoft Corporation, Redmond, WA) and SPSS software (IBM Corp., Armonk, NY). Tests were performed at the significance level of p < 0.05.

Each variable was coded numerically, 0/1 for no/yes, and 1/2/3/… for variables with several modalities. Quantitative variables were presented by arithmetic mean and standard deviation (mean ± SD), median, and interquartile range, while qualitative variables were presented by frequencies and percentages. The Kolmogorov-Smirnov normality test was applied to assess the distribution of the data. In addition, the chi^2^ test was used to compare two qualitative variables, and the Student's t test to compare the qualitative and quantitative variables.

Univariate and multivariate logistic regressions were used to highlight the associations between the variables. Thus, a multivariate regression model was built from the results of the univariate regression to test the relation between the MMTV-like virus status and several factors. However, the model included only the factors that had a p-value less than 15% in the univariate regression.

## Results

Description of the study population and the prevalence of MMTV-like virus in breast milk

The average age of the 44 lactating mothers who participated in this study was 27.11 ± 6.76 years, ranging between 18 and 43 years. Nursing women were mainly from urban areas (61.4%), 45.5% of them had previously breastfed their children (20/44), 52.3% (23/44) were primiparous, and 61.4% (27/44) of them delivered at term. The majority have reported a household income of less than 340$ (77.3%). The socio-demographic characteristics of the studied population are reported in Table 2.

**Table 2:** Association between maternal parameters and MMTV-like virus in breast milk among Moroccan lactating mothers. Statistical comparisons of age, height, weight, and BMI were performed using a t-test, while categorical variables were analyzed using the chi-square test. MMTV: mouse mammary tumor virus.

Variables related to the mothers	Number of participants (n = 44) (%)	Presence of MMTV-like virus		p (t-test)	p (Chi^2^ test)
		No (n = 24)	Yes (n = 20)		
Age (years)	27.11 ± 6.76	26.96 ± 7.15	27.30 ± 6.44	0.87	
Height (cm)	160 ± 6.025	161.96 ± 5.74	158.89 ± 6.07	0.10	
Weight before pregnancy (kg)	62.93 ± 9.258	62.39 ± 9.41	63.75 ± 9.37	0.70	
BMI	24.65 ± 3.52	23.72 ± 2.70	25.58 ± 4.33	0.16	
Area of residence					0.42
Rural	17 (38.6)	8 (33.3)	9 (45)		
Urban	27 (61.4)	16 (66.7)	11 (55)		
Parity					0.78
Primiparous	23 (52.3)	13 (54.2)	10 (50)		
Multiparous	21 (47.7)	11 (45.8)	10 (50)		
Previous breastfeeding					0.58
Never	24 (54.5)	14 (58.3)	10 (41.7)		
Yes	20 (45.5)	10 (50)	10 (50)		
Maternal education					0.42
Illiterate	16 (36.4)	9 (37.5)	7 (35)		
Elementary school	10 (22.7)	4 (16.7)	6 (30)		
Middle and high school	17 (38.6)	11 (45.8)	6 (30)		
University degree	1 (2.3)	0	1 (5)		
Total monthly family income					0.40
<350$	34 (77.3)	18 (75)	16 (80)		
350-529$	2 (4.5)	2 (8.3)	0		
530-999$	7 (15.9)	3 (12.5)	4 (20)		
˃1000$	1 (2.3)	1 (4.2)	0		

The β-globin gene was successfully amplified in all 44 samples, suggesting that DNA was correctly extracted from all samples and adequate for further analysis. PCR amplification that was performed using primers from the conserved region of the viral *env* gene DNA showed the presence of MMTV-like DNA in 45.5% of breast milk samples (20/44).

The results of the statistical tests showed no significant association between the presence of MMTV-like viral DNA in breast milk and anthropometric, sociodemographic, and economic variables (Table 2) as well as the mother's eating habits (Table 3).

**Table 3:** Association between maternal dietary and lifestyle factors and MMTV-like virus in breast milk among Moroccan lactating mothers. MMTV: mouse mammary tumor virus.

Variables	Number of participants (N = 44)	Presence of MMTV-like virus, n (%)		p (Chi^2^)
		No (n = 24)	Yes (n = 20)	
Red meat				0.12
Never	2	2 (8.3)	0	
Once a week	20	8 (33.3)	12 (60)	
Twice or more a week	22	14 (58.3)	8 (40)	
Poultry				0.52
Once a week	6	4 (16.7)	2 (10)	
Twice or more a week	38	20 (83.3)	18 (90)	
Canned food				0.25
Never	24	11 (45.8)	13 (65)	
Once a week	18	11 (45.8)	7 (35)	
Twice or more a week	2	2 (8.3)	0	
Wheat				0.88
Once a week	3	2 (8.3)	1 (5)	
Twice or more a week	8	4 (16.66)	4 (20)	
Every day	33	18 (75)	15 (75)	
Cereals (other than wheat)				0.15
Never	1	0	1 (5)	
Once a week	16	7 (29.2)	9 (45)	
Twice or more a week	23	13 (54.2)	10 (50)	
Every day	4	4 (16.7)	0	
Legumes				0.54
Once a week	22	11 (45.8)	11 (55)	
Twice or more a week	22	13 (54.2)	9 (45)	
Milk and dairy products				0.09
Once a week	3	3 (12.5)	0	
Twice or more a week	14	5 (20.8)	9 (45)	
Every day	27	16 (66.7)	11 (55)	
Tea				0.49
Never	2	2 (8.3)	0	
Once a week	7	3 (12.5)	4 (20)	
Twice or more a week	5	2 (8.3)	3 (15)	
Every day	30	17 (70.8)	13 (65)	
Coffee				0.97
Never	8	4 (16.7)	4 (20)	
Once a week	23	13 (54.2)	10 (50)	
Twice or more a week	5	3 (12.5)	2 (10)	
Every day	8	4 (16.7)	4 (20)	
Use of henna				0.03
Never	8	7 (29.2)	1 (5)	
Occasional	36	17 (70.8)	19 (95)	

Factors associated with the presence of MMTV-like virus in breast milk in Morocco

Chi^2^ and Student's T-test Results

A statistically significant association was found between the use of henna and the presence of the virus (p = 0.03). However, the statistical analysis did not demonstrate the influence of anthropometric, sociodemographic, clinico-obstetric, and economic parameters, as well as other dietary habits, on the presence of the MMTV-like virus in the breast milk of the participants may be due to the sample size. Tables 3, 4 present the results of the statistical tests.

**Table 4:** Results of simple and multiple logistic regression of the presence of MMTV-like virus in breast milk of lactating women from Morocco. MMTV: mouse mammary tumor virus.

Variables	Univariate analysis			Multivariate analysis		
	OR	95% CI	P-value	OR	95% CI	P-value
Age	1.008	0.922-1.101	0.866			
Height	0.913	0.819-1.019	0.099	0.942	0.832-1.066	0.340
Weight before pregnancy (kg)	1.016	0.938-1.101	0.689			
BMI	1.192	0.915-1.552	0.169			
Area of residence	0.610	0.180-2.077	0.430			
Parity	1.182	0.360-3.880	0.783			
Previous breastfeeding	1.400	0.422-4.623	0.581			
Maternal education	0.961	0.502-1.839	0.904			
Total monthly family income	0.920	0.450-1.879	0.818			
Red meat	0.744	0.267-2.072	0.572			
Poultry	1.800	0.294-11.031	0.525			
Canned food	0.424	0.141-1.278	0.128	0.703	0.187-2.637	0.601
Wheat	1.100	0.403-3.003	0.853			
Cereals (other than wheat)	0.348	0.125-0.972	0.044	0.273	0.061-1.219	0.089
Legumes	0.692	0.210-2.280	0.545			
Milk and dairy products	1.022	0.392-2.663	0.965			
Tea	1.041	0.543-1.995	0.904			
Coffee	1.009	0.546-1.863	0.977			
Use of henna	7.824	0.871-70.261	0.066	11.549	1.034-129.005	0.047

Binary Logistic Regression Results

The results of univariate logistic regression analysis showed a significant association between the presence of MMTV-like viral DNA in breast milk and the frequency of cereal consumption by the mothers (OR = 0.348, p = 0.044).

Indeed, all variables that had a p-value <20% were included in the multivariate model. The results revealed significant associations between the presence of the MMTV-like viral DNA in the breast milk of Moroccan lactating women and the use of henna (OR = 11.549; p = 0.047). The use of henna by women increases the risk of the presence of MMTV-like viral DNA in breast milk (95% CI: 1.034-129.005). Table 4 presents the results of the binary logistic regression.

## Discussion

This study shows the occurrence of MMTV-like virus in 20/44 (45.5%) of milk samples among healthy nursing mothers from Rabat, the capital of Morocco. This prevalence is higher than the findings of the only two studies conducted in this context so far.

The first one was carried out in Australia by Johal et al. [20] on 91 healthy breastfeeding women and revealed the presence of the MMTV-like sequence in 5% of the analyzed breast milk samples. The second one was by Nartey et al. [16], who performed a study on breast milk samples within American healthy breastfeeding women as a reference group, and breastfeeding women who underwent breast biopsy, with positive results for both groups but with a higher rate in the biopsy group (7.6% vs. 20.5%). They concluded the possibility of the transmission of the MMTV-like sequence in humans.

Unfortunately, the aforementioned studies focused on the detection and prevalence of MMTV-like viral DNA in breast milk and did not investigate the associated factors, limiting the resources to compare and discuss our statistical results.

However, a Moroccan study performed in 2014 among 42 Moroccan patients suffering from breast cancer revealed the presence of MMTV-like *env* sequences in 57.14% of the analyzed samples, which coincides with our finding. In addition, the study reported a significant association between the presence of MMTV sequences and parity [17], but our study did not find any association between parity and the presence of the virus in the breast milk of the participants.

In the present study, a significant association with the use of henna by the mothers was found. There is a possible association between the use of henna and the risk of the presence of MMTV-like virus in breast milk. The hypothesis could be the possible contamination of the henna (leaves and powder) sold in bulk in local markets by mice urine and feces during storage.

In fact, zoonotic transmission (from animals to humans) of MMTV is possible. The particles in suspension on the surfaces used by mice are household allergens; therefore, they are theoretical sources of transmission of MMTV from mice to humans (possibly by contact and/or by ingestion). The transmission of the MMTV to humans through ingestion of food contaminated with mouse feces and urine might be possible. Further, MMTV gene sequences were detected in human saliva too, which could be a route in inter-human infection [21]. Other studies are in favor of the transmission of this virus by domestic animals such as dogs and cats [22,23]. Humans could attain MMTV-like virus through contact with their infected pets [5]. Women with companion dogs are at twice the expected risk of breast cancer. These observations are suggestive of transmission of MMTV in dog saliva to humans [24]. This could explain the importance of its detection in Western countries where the dog is the most common domestic animal.

Stewart and colleagues suggested that the global distribution of MMTV-like virus sequences in breast cancer among human populations correlates with the distribution of its natural host, the house mouse (*Mus musculus domesticus*) [25]. Their research highlighted that countries with the highest breast cancer incidence also have a predominance of *Mus domesticus*. For instance, in Southern California, exogenous MMTV sequences were detected in 50% of the examined *Mus domesticus* population. These findings led Stewart et al. to hypothesize that a high concentration of *Mus domesticus* may be associated with increased MMTV prevalence. Given that *Mus musculus domesticus* is also the dominant species in Morocco [26], it is plausible that this mouse population serves as a reservoir for the virus in the region, emphasizing the need for further research to detect and study the virus in Moroccan mice.

More recently, Lehrer and Rheinstein proposed that MMTV transmission from mice to humans might explain the observed association between breast cancer and higher socioeconomic status. They suggested that girls from wealthier families, who often grow up in clean and well-maintained homes, may experience delayed exposure to *Mus domesticus*, impacting infection dynamics [27]. Additionally, Stewart and Chen revisited the zoonotic transmission hypothesis, noting that the globalization of *Mus domesticus* has expanded its range to new regions, potentially contributing to rising breast cancer rates in Asia [28]. These studies collectively underscore the importance of understanding MMTV's zoonotic potential and its environmental and socioeconomic implications.

However, our study does have certain limitations, primarily related to sampling. The relatively small number of participants may have influenced the number of associations observed.

## Conclusions

The present study clearly showed the presence of MMTV-like DNA in breast milk samples with a high frequency, reinforcing the possible association between these viruses and breast cancer and opening a new gate for better management of this disease. This study prompts conducting further evaluations on large samples to elucidate the probable causal roles of MMTV-like virus in breast cancer development. In addition, studies concerning the detection of MMTV in mice in Morocco are necessary to highlight the sources and potential factors of exposure of the general population and women in particular to MMTV.
